# Siglec-H-Deficient Mice Show Enhanced Type I IFN Responses, but Do Not Develop Autoimmunity After Influenza or LCMV Infections

**DOI:** 10.3389/fimmu.2021.698420

**Published:** 2021-08-23

**Authors:** Nadine Szumilas, Odilia B. J. Corneth, Christian H. K. Lehmann, Heike Schmitt, Svenia Cunz, Jolie G. Cullen, Talyn Chu, Anita Marosan, Attila Mócsai, Vladimir Benes, Dietmar Zehn, Diana Dudziak, Rudi W. Hendriks, Lars Nitschke

**Affiliations:** ^1^Division of Genetics, Department of Biology, University of Erlangen-Nürnberg, Erlangen, Germany; ^2^Department of Pulmonary Medicine, Erasmus MC, University Medical Center, Rotterdam, Netherlands; ^3^Laboratory of Dendritic Cell Biology, Department of Dermatology, University Hospital Erlangen, Erlangen, Germany; ^4^Deutsches Zentrum Immuntherapie (DZI), University Hospital Erlangen, University of Erlangen-Nürnberg, Erlangen, Germany; ^5^Medical Immunology Campus Erlangen (MICE), University of Erlangen-Nürnberg, Erlangen, Germany; ^6^First Department of Medicine, University Hospital Erlangen, Erlangen, Germany; ^7^Division of Animal Physiology and Immunology, School of Life Sciences Weihenstephan, Technical University of Munich, Freising, Germany; ^8^Department of Immune Modulation, University Hospital Erlangen, Erlangen, Germany; ^9^Semmelweis University School of Medicine, Budapest, Hungary; ^10^Genomics Core Facility, EMBL Heidelberg, Heidelberg, Germany

**Keywords:** plasmacytoid dendritic cells, Interferon-alpha, Siglec, TLR9, SLE

## Abstract

Siglec-H is a DAP12-associated receptor on plasmacytoid dendritic cells (pDCs) and microglia. Siglec-H inhibits TLR9-induced IFN-α production by pDCs. Previously, it was found that Siglec-H-deficient mice develop a lupus-like severe autoimmune disease after persistent murine cytomegalovirus (mCMV) infection. This was due to enhanced type I interferon responses, including IFN-α. Here we examined, whether other virus infections can also induce autoimmunity in Siglec-H-deficient mice. To this end we infected Siglec-H-deficient mice with influenza virus or with Lymphocytic Choriomeningitis virus (LCMV) clone 13. With both types of viruses we did not observe induction of autoimmune disease in Siglec-H-deficient mice. This can be explained by the fact that both types of viruses are ssRNA viruses that engage TLR7, rather than TLR9. Also, Influenza causes an acute infection that is rapidly cleared and the chronicity of LCMV clone 13 may not be sufficient and may rather suppress pDC functions. Siglec-H inhibited exclusively TLR-9 driven type I interferon responses, but did not affect type II or type III interferon production by pDCs. Siglec-H-deficient pDCs showed impaired Hck expression, which is a Src-family kinase expressed in myeloid cells, and downmodulation of the chemokine receptor CCR9, that has important functions for pDCs. Accordingly, Siglec-H-deficient pDCs showed impaired migration towards the CCR9 ligand CCL25. Furthermore, autoimmune-related genes such as Klk1 and DNase1l3 are downregulated in Siglec-H-deficient pDCs as well. From these findings we conclude that Siglec-H controls TLR-9-dependent, but not TLR-7 dependent inflammatory responses after virus infections and regulates chemokine responsiveness of pDCs.

## Introduction

Systemic lupus erythematosus (SLE) is a systemic autoimmune disease with a complex pathogenesis and is characterized by the presence of autoantibodies, immune complex deposition in several tissues and kidney nephritis (1, 2). Besides a gender bias for women, SLE has a strong heritability with complex multigenetic factors, which affect the susceptibility of the disease. Various genome-wide association studies revealed more than 25 loci that are associated with susceptibility of SLE. These loci comprise genes that are involved in lymphocyte signalling, immune complex clearance, TLR pathways or type I interferon (IFN) production (3, 4). Type I IFNs have been linked to the pathophysiology of SLE. Accordingly, SLE patients with active disease show a type I IFN signature (5, 6). Typical autoantibodies in SLE are directed against DNA, RNA or nuclear antigens. Pattern recognition receptors of the TLR family including TLR7 and TLR9, that bind single-stranded RNA, or DNA, respectively, can be activated not only by bacterial or viral DNA or RNA, but also by immune complexes containing endogenous nucleic acids (7, 8). The activation of TLR7 or TLR9 by immune complexes that contain self-proteins and DNA or RNA leads to the induction of type I IFN. Such immune complexes containing autoantibodies may be present in SLE due to defective clearance of apoptotic cells (9). The type of immune cells that generate most type I IFN are plasmacytoid dendritic cells (pDCs). pDCs can secrete large amounts of type I IFNs, like IFN-α or IFN-β , upon stimulation of their endosomal TLR7 or TLR9 or upon stimulation of cytoplasmic nucleic acid sensors (10, 11).

An efficient host defence against viruses depends on a strong IFN-α response (12, 13). As pDCs constitutively express TLR7 and TLR9, virus RNA or DNA can bind to these TLRs, respectively, and trigger type I IFN production by these cells (14). pDCs are critical for induction of high type I IFN levels at an early phase after a virus infection. Besides type I IFN, pDCs are also known to generate high levels of type III IFN (IFN-λ) in response to viral infections (15). Because most SLE patients have a type I IFN signature, it is controversially discussed whether previous virus infections that led to type I IFN production may be involved in triggering this autoimmune disease. Viral infections such as EBV or hepatitis B virus can trigger transient autoantibody production, but whether this can lead to SLE is unclear (16–18). We have previously shown that murine cytomegalovirus (mCMV) infection in Sialic-acid-binding Ig-type lectin H (Siglec-H) knockout (ko) mice can trigger a fulminant lupus-like autoimmune disease several weeks later (19). Siglec-H is a cell surface protein on pDCs, which acts as a negative regulator of type I IFN responses. mCMV-infected Siglec-H ko mice showed an enhanced IFN-α response, but still controlled the virus normally. Several weeks after the mCMV infection Siglec-H ko mice developed high levels of autoantibodies, accompanied by a strong kidney nephritis. This development of autoimmune disease after virus infection led to a strong type I IFN signature and was dependent on type I IFN signalling, as demonstrated by the lack of virus-induced disease in type I IFN receptor (IFNAR) ko mice (19).

Siglec-H is expressed in a quite restricted fashion on mouse pDCs, a subset of marginal zone macrophages in the spleen, on medullary macrophages in the lymph node and on microglia in the brain (20–22). Most Siglec receptors are inhibitory receptors, which inhibit responses of activating receptors by recruitment of phosphatases to immunoreceptor tyrosine-based inhibition motifs (ITIMs). Siglec-H lacks an ITIM motif, but is associated with DAP12 on the pDC surface (20). Several studies showed that Siglec-H-deficient pDCs produce more IFN-α after TLR9 stimulation *in vitro*, or after mCMV infection *in vivo* (19, 23, 24). How this inhibition of IFN-α production is achieved by Siglec-H is unclear. But it is clear that the association of Siglec-H with DAP12 is crucial. Although DAP12 has two activating ITAM motifs in its intracellular tail and can recruit Syk, paradoxically it inhibits TLR-induced signalling on pDCs (25, 26). Also, DAP12-deficient pDCs lack Siglec-H surface expression and produce higher amounts of IFN-α after CpG stimulation (27). This links the inhibitory function of Siglec-H on pDCs to DAP12.

mCMV is a persistent DNA virus, which is controlled in mice by the immune system, but cannot be cleared after the infection. Our result, that mCMV can trigger an autoimmune disease in a mouse strain that produces more type I IFN (19) led to the question, whether this finding is restricted to mCMV, or can be extended to other virus infections. In particular, the question arose whether non-persistent virus infections, or other types of viruses, like RNA viruses can also trigger a lupus-like autoimmune disease in Siglec-H ko mice. We therefore infected Siglec-H ko mice with influenza virus (an ssRNA virus of the *Orthomyxoviridae* family), which is rapidly cleared by the immune system, and with Lymphocytic Choriomeningitis virus (LCMV) clone 13, a ssRNA virus of the *Arenaviridae* family, which leads to a chronic infection in the mouse. After infections with both types of viruses, we observed the mice for several weeks for signs of autoimmunity. Furthermore, we analysed the changes in the gene expression pattern of Siglec-H-deficient pDCs in order to analyse the regulation of type I IFN responses by Siglec-H in this cell type.

## Materials And Methods

### Mice

Siglec-H ko mice on a C57BL/6 background were obtained from the Consortium for Functional Glycomics by a gift from J. Paulson (Scripps Research Institute, La Jolla, CA) (28). Wild type (wt) and Siglec-H ko mice were littermates of heterozygous breeding pairs. Bones of Hck ko and Hck/Fgr/Lyn ko mice (29) were obtained from A. Mócsai (Semmelweis-University, Budapest). Animal experiments were performed in accordance with the German law for protection of animals, after approval by the animal welfare committee (Regierung von Unterfranken).

### Virus Infection Models

For influenza infection wt and Siglec-H ko mice (8-12 weeks, both sexes) were infected intranasally with 1 x 10^4^ pfu of H3N2 X-31 influenza (30). To monitor the infection mice were weighed daily until 10 days pi. For the short-term experiment mice were analysed on day 10 pi. In order to evaluate a possible induction of autoimmunity infected mice were kept for 20 weeks and blood for sera was drawn every 4 weeks. To investigate the influence of LCMV in wt and Siglec-H ko mice, LCMV-cl13 strain was used. Mice (8-12 weeks, both sexes) were infected with 5 x10^6^ pfu of LCMV-cl13 i.v. and monitored over a period of 35 weeks. Blood was drawn every 4 weeks.

### Quantification of Influenza Specific Antibodies

Quantification of influenza X31 specific antibodies in sera of infected mice 20 weeks pi was performed exactly as described (30).

### FACS Analysis

Single-cell suspensions of bone marrow, spleen, and lymph nodes were incubated with 1 x ACK solution (for red blood cell lysis) to deplete erythrocytes. Staining was performed with the following antibodies (conjugated with FITC, PE, APC, eFI450, BV786, AF700, PE-Cy7, PerCP Cy5.5, APC-eFI780 or biotin): anti–Siglec-H (551.3D3; Biolegend), anti-mPDCA1 (JF05-1C24.1; Biolegend), anti-CD11c (N418; Biolegend), anti-B220 (RA3-6B2; Biolegend), anti-CCR9 (242503; R&D Systems), anti-FcgR2b (own hybridoma), anti-IL2r (PC61; Biolegend), CD19 (eBio1D3, eBioscience), CD3 (17A2, eBioscience), CD5 (53-7.3, eBioscience), CD4 (GK1.5, eBioscience), CD138 (281-2, BD), CD95 (Jo2, BD), IgD (11-26c.2a, BD), CD80 (16-10A1, eBioscience), PD-L2 (TY25, BD), IL-4 (11B11, BD), IL-6 (MP5-20F3, BD), IL-10 (JES5-16E3, eBioscience), IFN-g (XMG1.2, eBioscience) and Fc-block (2.4G2; own hybridoma). Biotinylated antibodies were detected using streptavidin PerCPCy5.5 or streptavidin PECy7 (Biolegend). Cells were analysed using either Cytoflex (Beckmann Coultier) and FlowJo software (Tree Star).

### Preparation of Lymphocytes From the Intestine

The protocol of “Lamia propia dissociation kit” from Miltyenyi Biotec was followed in all details. In brief, the lower intestine or the colon was removed from sacrificed mice, the feces were removed by flushing with HBSS and then the tissue was cut in pieces. The protocol was followed using MACS mix Tube Rotators and MACs SmartStrainers. To remove erythrocytes and dead cells a Percoll density gradient centrifugation was done and the lymphocyte band was collected.

### Detection of ANAs Using HEp-2 Cells

Antinuclear antibodies (ANAs) were detected by incubating a 1:250 serum dilution on HEp-2 cells (Immco Diagnostics). After 30-min incubation at room temperature, antinuclear total IgG was detected by Alexa Fluor 488–conjugated goat anti–mouse Fcγ;-specific IgG (Jackson Immunoresearch). Slides were analysed with a 10x magnification on a fluorescence microscope. A serum pool of MRL/lpr mice (3-4 months old) was used as positive control. ImageJ64 (National Institutes of Health) was used to determine the fluorescence intensity.

### ELISA Assay for dsDNA Autoantibodies

Detection of dsDNA autoantibodies in mouse sera was performed as described (19). In Short: 0.01% Poly-L-lysine (Sigma-Alderich) were used to precoat MaxiSorp plates (Nunc). After 2 h at RT, plates were coated with dsDNA from calf thymus (20µg/ml; Sigma-Aldrich) in H_2_0 over night at 4°C. Sera were added in 1/3 serial dilutions starting at 1/20, pooled serum of SLE affected MRL/lpr mice, with a starting dilution of 1/250, served as standard. Goat-anti-mouse IgG (1 mg/ml) coupled to AP was used for detection.

### Blood Urea Nitrogen (BUN)

In order to analyse the urea nitrogen content in blood as a sign for kidney damage the enzymatic BUN kit from TECODiagnostics was used. Therefore, sera were diluted 1:100 in enzymatic reagent on 96-well plates. After incubation with colour reagent, urea nitrogen content was measured at a 600-nm wavelength with the ELISA reader (VersaMaxPLUS; Molecular Devices). Values <20 mg/dl were considered as normal.

### Generation of BM-Derived pDCs

BM cells were isolated from femur and tibia of wt, Siglec-H ko, Hck ko or Hck/Fgr/Lyn ko mice and erythrocyte lysis was performed with 1 x ACK solution. BM cells were then cultured for 8 days at a concentration of 2 x 10^6^ cells/ml in complete RPMI medium [4 mM L-glutamine, 100 U/ml penicillin, 100 μg/ml streptomycin, 10 mM HEPES, 50 µM 2-mercaptoethanol, 100µM nonessential amino acids, 1 mM sodium pyruvate, and 10% FCS with 50 ng/ml rmFlt3L (Biolegend)] in 25-cm^2^ cell culture flasks (each with 5 ml of cell suspension). On d4 of culture half of medium per flask was replaced by fresh medium containing 50 ng/ml rmFlt3L. After d8, CD11c^+^PDCA^+^ pDCs were purified with magnetic-activated cell-sorting separation columns using mouse pDC isolation Kit (Miltenyi Biotec).

### *In Vitro* Stimulation of pDCs

*In vitro* generated pDCs were cultured in 96-well plates (10^5^ cells/well in 250 µl) using complete RPMI and were stimulated for 24 h with different stimuli: 2 – 5 µg/ml CpG-A oligodeoxynucleotide 1585 (InvivoGen), 2 µg/ml LPS (Invivogen), 2 – 5 µg/ml R848 (Invivogen) or 2 – 10 µg/ml 3’3’-cGAMP (Invivogen). Supernatants were used for cytokine quantification.

### Quantification of Cytokines

To measure the amount of produced cytokines in supernatants of *in vitro* pDC cultures cytokine ELISAs were performed. For detection of IFNα the protocol described in (19) was used. In short: To detect IFNα by ELISA, maxisorp plates (Nunc) were coated with anti-mouse IFNα (clone: RMMA-1, PBL Interferon Source) A polyclonal anti-IFNα antibody from rabbit (PBL Interferon Source) and a HRP conjugated anti-rabbit IgG (H+L) antibody (Dianova) served as secondary reagent. For detection of IFNλ (IL28/29) the mouse IL-28B/IFN-lambda 3 DuoSet ELISA kit (R&D Systems) was used according to manufacturer’s instructions.

### Preparation of cDNA Library for RNA Sequencing

For the gene expression analysis of naive wt and Siglec-H ko pDCs single cell suspensions of splenocytes were sorted for pDCs (B220^+^CD19^-^CD11b^-^CD11c^+^PDCA-1^+^). Sorted cells were directly collected into RLT-lysis buffer with 1% 2-mercaptoethanol. RNA isolation was performed with RNeasy Micro Plus kit (QIAGEN) according to manufacturer’s instructions. A quality check of the isolated RNA was performed using the Experion RNA HighSens lab-on-a-chip system. RNA samples were sent to EMBL institute in Heidelberg for RNA sequencing.

### Gene Expression by Quantitative Realtime PCR (qRT-PCR)

pDCs were isolated from spleen using MACS and PDCA-1-beads (Miltenyi) and RNA was isolated by using RNeasy Micro Plus Kit (QIAGEN). cDNA was synthesized using a reverse transcriptase system (FastGene Scriptase II kit; Nippon Genetics). qRT-PCR was performed with a SYBR Green based kit (QuantiNova SYBRGreen Kit; QIAGEN) on a AriaMx Realtime machine (Agilent). Actin was used as the housekeeping gene. The following primers were used:

Actin fw, 5’ CCA ACT GGG ACG ACA TGG AG 3’
Actin rev, 5’ CTC GTA GAT GGG CAC AGT GTG 3’
IL2R fw, 5’ CCACATTCAAAGCCCTCTCCTA 3’
IL2R rev, 5’ GTTTTCCCACACTTCATCTTGC 3’
Fcgr2b fw, 5’ TGTCCAAGCTCCCAACTCTTCACC 3’
Fcgr2b rev, 5’ GTGTTCTCAGCCCCAACTTTG 3’
Dnase1l3 fw, 5’ TCC CCT TGC ACA CAA CTC CCG A 3’
Dnase1l3 rev, 5’ AAC GGG GAA CCA CGG AGT TGA C 3’
CD4 fw, 5’ TCC TTC CCA CTC AAC TTT GC 3’
CD4 rev, 5’ AAG CGA GAC CTG GGG TAT CT 3’
Klk1 fw, 5’ CCGCTTCACCAAATATCAATGTG 3’
Klk1 rev, 5’ GCTCATCTGGGTATTCATATTTGACG 3
CCR9 fw, 5’ CAA TCT GGG ATG AGC CTA AAC AAC 3’
CCR9 rev, 5’ ACC AAA AAC CAA CTG CTG CG 3’
Hck fw, 5’ GTGAAGTCCAGGTTCCTCCG 3’
Hck rev, 5’ ACCACAATGGTATCCTCAGAGC3’
Cldn1 fw, 5’ CTGGAAGATGATGAGGTGCAGAAGA 3’
Cldn1 rev, 5’ CCACTAATGTCGCCAGACCTGAA 3’
CD11c fw, 5’ CTGGATAGCCTTTCTTCTGCTG 3’
CD11c rev, 5’ GCACACTGTGTCCGAACTC 3’
mDOCK4 fw, 5’ GGT ATA TTT CCT TCC AGC TAT G 3’
mDOCK4 rev, 5’ GAC ATA GAG CTG TTT CCA CAT G 3’
mPmepa1a fw, 5’ GAA GGA TGC CTC TGG CCC T 3’
mPmepa1a rev, 5’ CCAGCTGTTGCTCAGGGTC 3’
Sdc2 fw, 5′ CTG GCC ACC GAC TAT GAG AA 3’
Sdc2 rev, 5′ AAA ATC CAC GTG AAA AAG TTG GA 3’
CD244a fw, 5’ GTTGCCACAGCAGACTTTC 3’
CD244a rev, 3’ TTCCAAC CTCCTCGTACACGGTAC 3’

### Transwell Migration Assay

pDCs of spleens from B16-Flt3L injected mice were sorted and used in a transwell migration assay to analyse their migration capacity towards specific chemokines. Transwell inserts (6,5 mm insert, 5.0 μm polystyrene-membrane; costar^®^) were placed in 24 well plates and 5 x10^4^ sorted pDCs were added into the insert in a total volume of 100 µl medium. The lower chamber was filled with 600 µl of medium including the specific chemokine ligand: CCL25 (125 nM, Biolegend) or CXCL13 (100 nM, Biolegend). The assay was incubated for 2 h at 37°C and cells in the lower chamber were counted three times independently. The ratio of migrated cells towards the individual non-chemokine control was calculated.

### Statistics

Prism 7 (GraphPad Software) was used to calculate the statistics for all graphs shown. After testing for normal distribution, significant differences between samples were calculated using unpaired Student’s *t* test or Mann-Whitney U-rank test.

## Results

### Influenza Infection of Siglec-H ko Mice Leads to an Overall Similar Virus-Specific Immune Response as in wt Mice and Does Not Induce Autoimmunity

**Figure 1:**
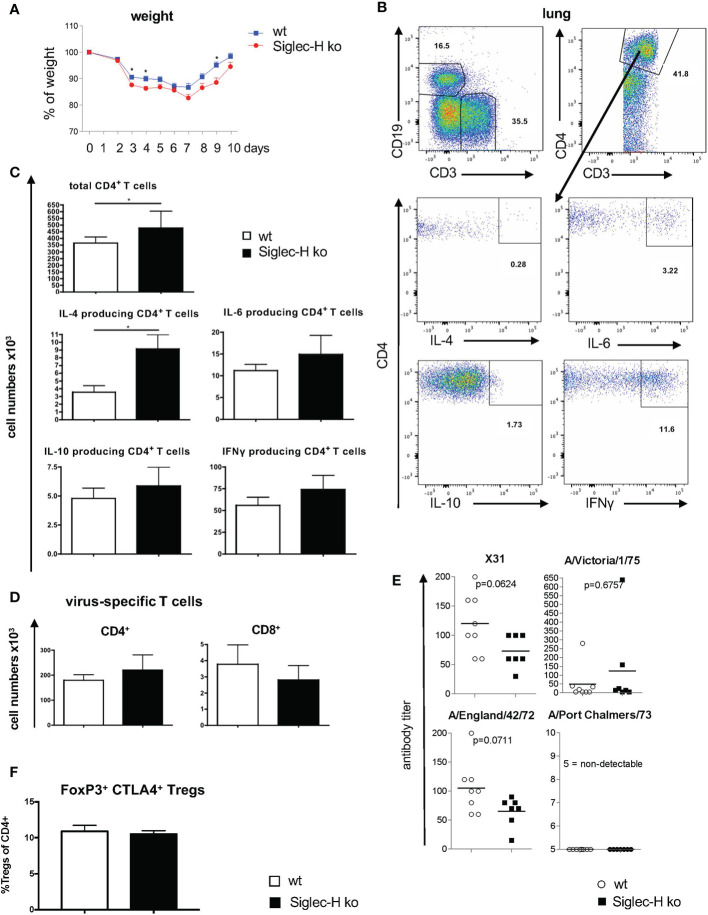
Increased weight loss and more CD4+ T cells in lungs of Siglec-H ko mice 10 days after influenza H3N2 infection. wt and Siglec-H ko mice were intranasally infected with 10^4^ pfu Influenza H3N2 (X-31) virus. On day 10 the mice were euthanized and cell populations were analysed *via* flow cytometry. Serum was analysed on virus-specific antibodies after 20w. **(A)** Weight loss of wt and Siglec-H ko mice over 10 days of Influenza infection. **(B)** Gating strategy for the analysis of intracellular cytokines in CD4^+^ T cells. Living, single lymphocytes were stained with anti-CD3 and anti-CD19 to select T and B cells. CD3^+^ cells were marked as T cells and subsequently divided by CD4 and CD8 expression. Intracellular cytokines IL-4, IL-6, IL-10 and IFNy were stained in CD4+ and CD8+ T cells. **(C)** Absolute numbers (x10^3^) of total CD4+ T cells and IL-4, IL-6, IL-10 and IFNγ; producing CD4 T cells in lungs of wt and Siglec-H KO mice. **(D)** Absolute numbers (x10^3^) virus-specific CD4 and CD8 T cells analysed by fluorescence-coupled Influenza peptide ASNETMN staining. **(E)** Serum titers of strain-specific antibodies in wt and Siglec-H ko mice 20 weeks p.i. **(F)** Percentages of CD4+ FoxP3+ CTLA4+ Tregs in medestrial lymph nodes, 10 days after influenza infection. Mice were aged between 8-14 weeks, Data from one experiment, N=10 per genotype. Mann-Whitney test, *p < 0.05. Error bars are mean ± SD values.

As a mCMV virus infection triggered a severe autoimmune disease in Siglec-H ko mice (19), we wanted to extend our studies to other types of viruses. First we infected Siglec-H ko and wt mice with influenza virus A H3N2 intranasally. Influenza virus triggers an acute infection, which is rapidly cleared by the immune system and which is non-persistent. Nevertheless, after inflammation is resolved, B and T cells assemble into organized structures known as inducible bronchus-associated lymphoid tissue. We first performed a short-term experiment to examine the virus infection during the initial phase. We followed the weight loss during the first 10 days of the infection. We observed weight loss until day 7 in both groups of mice and then a regain of weight from day 8 onwards. Siglec-H ko mice showed a stronger weight loss with significant differences at days 3, 4 and 9, indicating a more severe course of the infection in this mouse strain (**Figure 1A**). We sacrificed the mice at day 10 and analysed the immune cell composition in the lung and in lymphoid organs. We did not observe significant differences in pDC numbers in bone marrow or lung between Siglec-H ko and wt mice (**Supplementary Figures 1A, B**). pDCs were analysed, as they are the main immune cell population expressing Siglec-H. Next, we analysed B cell infiltration into the lung and determined intracellular cytokine production by B cells, but did not find any changed cytokine production (**Supplementary Figures 1C, D**). There was a higher number of lung infiltrating CD4+ T cells after influenza infection of Siglec-H ko mice compared to wt mice and among these a higher IL-4 production (**Figures 1B, C**). Virus-specific T cells in the lung were determined by staining with an influenza-peptide tetramer staining and no differences were found between infected Siglec-H ko and wt mice (**Figure 1D**). Also, we did not detect a difference of Tregs in the medestrial lymph nodes, 10 days after influenza infection (**Figure 1F**).

In order to follow the induction of a possible autoimmune disease after virus infection, in a second set of experiments mice were infected with influenza A H3N2 intranasally and analysed 7 weeks and 20 weeks after infection. After 20 weeks the immune status of the mice was analysed. B cell and T cell subpopulations were examined in the lung and in the spleen. There were no significant differences detected in B- or T-cell sub-populations nor in cytokine secretion in the lung. The only difference was a lower number of naïve T cells and a higher number of activated T cells in spleen and lung of Siglec-H ko mice (**Supplementary Figures 2** and **3**). Virus-specific antibody serum titres against the used virus strain (X31) and cross-reacting antibodies against 3 other virus strains were determined on week 20 by a hemagglutinin inhibition assay and no significant differences in virus-specific antibody responses were detected between the two mouse groups (**Figure 1E**). In conclusion the influenza virus infection led to a somewhat more severe early phase of the infection, but triggered an overall similar virus-specific immune response.

**Figure 2:**
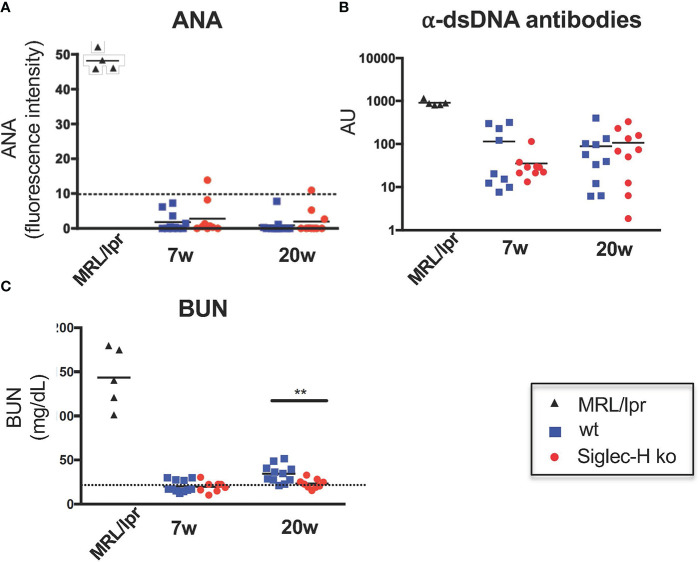
No signs of autoimmunity in Siglec-H ko mice upon influenza infection. Mice were infected with Influenza virus H3N2 (10^4^ pfu) and sera were analysed for the presence of ANAs, anti-dsDNA antibodies or BUN (blood urea nitrogen). Sera of influenza infected mice were semi-quantitatively analysed on HEp-2 slides **(A)**. The mean pixel intensity was determined from 10 randomly analysed cells per sample. Each symbol represents the IgG serum ANA level of a single mouse. Representative of one experiment (n = 10 and n = 5 for MRL/*lpr*). **(B)** Anti-dsDNA antibodies in sera of influenza-infected mice were measured by ELISA. Each symbol represents a single mouse. AU, arbitrary units. **(C)** Urea nitrogen contents in the blood of influenza-infected mice were determined with an enzymatic BUN kit. Representative of one experiment (n = 10 mice and n = 5 MRL/lpr mice). **p < 0.01; Mann-Whitney test. Sera of MRL/lpr mice served as a positive control.

Our main aim of the influenza infection experiments was to investigate whether autoimmunity is induced in Siglec-H ko mice, as we had observed after mCMV infections. Therefore, we determined antinuclear antibodies (ANA), as well as anti-dsDNA antibodies 7 weeks and 20 weeks after influenza infection. We did not detect significant levels of ANA or anti-dsDNA antibodies in Siglec-H ko mice or in wt controls (**Figures 2A, B**). Furthermore, blood urea nitrogen levels (BUN) were determined in the serum of infected mice. A rise in BUN indicates kidney dysfunction, as is typical for SLE and as it was found in the positive control MRL/lpr. No increased BUN was detected in Siglec-H ko mice or in wt controls up to 20 weeks after the infection, there was even a significantly lower BUN level in Siglec-H ko mice 20 weeks after infection. (**Figure 2C**). Taken together, from these findings we conclude that in contrast to mCMV, influenza infection did not induce autoimmunity in Siglec-H ko mice.

### LCMV Clone 13 Infections Resulted in a Similar Weight Loss and Did Not Lead to Differences in the Induced Autoantibody Responses Between Siglec-H ko and wt Mice

**Figure 3:**
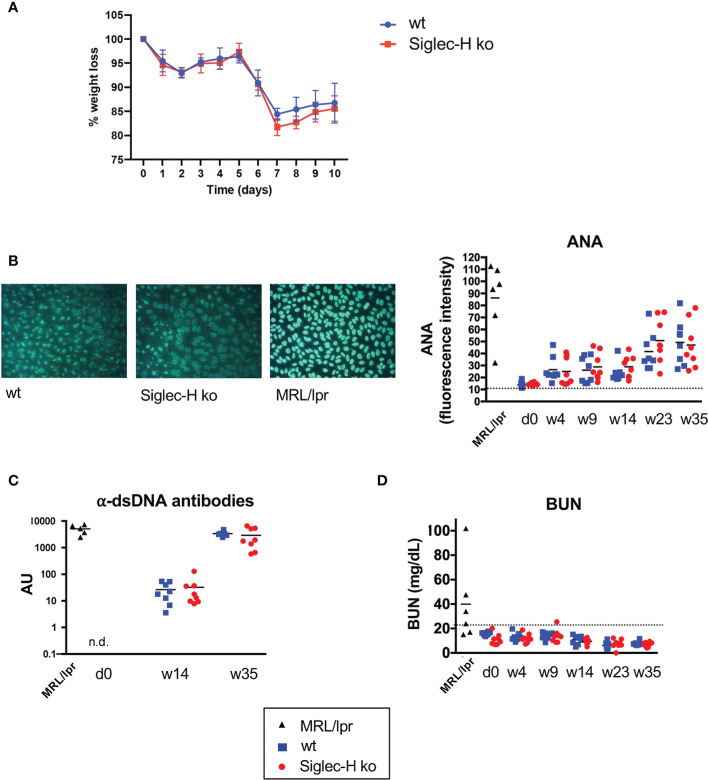
After LCMV clone 13 infection Siglec-H ko mice show less weight loss and no increased autoantibody levels compared to wt mice. Mice were infected with LCMV clone 13 virus (5 x 10^6^ pfu). **(A)** Weight loss of wt and Siglec-H ko mice over 10 days of LCMV infection. **(B)** Sera of LCMV-infected mice were semi-quantitatively analysed on HEp-2 slides for ANA antibodies. On the left example pictures from HEp-2 stainings of week 35 are shown. The mean pixel intensity was determined from 10 randomly analysed cells per sample and is shown on the right. Each symbol represents the IgG serum ANA level of a single mouse. **(C)** Anti-dsDNA antibodies in sera of LCMV-infected mice were measured by ELISA. Each symbol represents a single mouse. AU, arbitrary units. **(D)** Urea nitrogen contents in the blood of influenza-infected or LCMV-infected mice were determined with an enzymatic BUN kit. Representative of two experiments (n = 8 mice and n = 5 MRL/lpr mice). Sera of MRL/lpr mice served as a positive control.

In order to examine another virus infection, which also induces a chronic infection, we chose LCMV. For this we used clone 13, a substrain of LCMV which causes a chronic infection compared to the parenteral LCMV Armstrong strain that is rapidly resolved (31, 32). We infected wt and Siglec-H ko mice intravenously and followed them for 35 weeks after the infection. To follow the severity of the infection, we determined the weight of the animals over the first 10 days post infection. A similar weight loss was detected in Siglec-H KO compared to wt mice (**Figure 3A**). The induction of Treg cells in the blood at 21 days after the LCMV infection was similar (**Supplementary Figure 4A**). We took blood every 4 weeks and followed ANA and anti-DNA antibodies up to week 35. Although we observed rising autoantibody levels in both groups of mice (particularly increasing anti-dsDNA antibodies), there was no difference between the wt and Siglec-H ko mice (**Figures 3B, C**). The ANA antibodies levels observed in wt and Siglec-H ko mice were weaker than in lupus-prone MRL/lpr mice and did not stain whole nuclei as in MRL/lpr mice, but stained nuclei with a more speckled pattern (**Figure 3B**). There was no difference in the staining pattern observed between wt and Siglec-H ko mice. BUN levels were followed as well and no increased BUN was determined over the whole time period (**Figure 3D**). Although the more chronic LCMV clone 13 infection seems to induce autoantibodies, there are no signs of autoimmune disease, as indicated by kidney dysfunction. Furthermore, also in this virus infection model Siglec-H ko mice did not show any signs of an induced autoimmune disease, as after mCMV infection.

### Siglec-H Does Not Inhibit IFN-λResponses by pDCs

**Figure 4:**
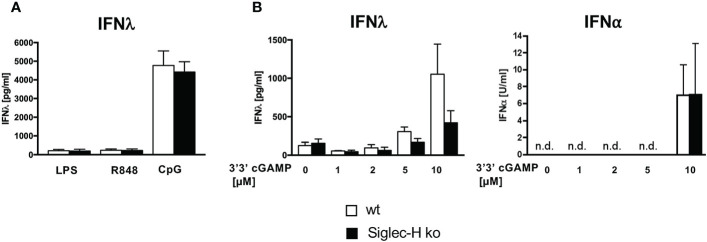
Siglec-H deficiency has no influence on the production of IFN-λ. *In vitro* generated pDCs were stimulated with different stimuli for 24 hrs and supernatants were analysed *via* ELISA for IFN-λand IFN-α. **(A)** *in vitro* generated pDCs stimulated with LPS (c=1 µg/ml), R848 (c=4 µg/ml) or CpG (c=2,5 µg/ml) for 24hrs. The amount of IFN-λ was measured *via* ELISA (R&D Systems). **(B)** *In vitro* generated pDCs were stimulated with different concentrations of 3’3’-cGAMP and amounts of both IFN-λ and IFN-⍺ were measured *via* ELISA. White bars: wt, black bars: Siglec-H ko. Summary of three independent experiments, n.d., not detectable. N=5-7 mice per genotype. Error bars are mean ± SD values.

We observed previously that CpG stimulation or stimulation with mCMV *in vitro* induced increased IFN-α production by Siglec-H-deficient pDCs. However, type II IFN (IFN-γ) production by Siglec-H-deficient pDCs was not increased after these stimulations (19). pDCs can also produce type III interferon (IFN-λ), which is also elevated in SLE patients (33, 34). We therefore determined IFN-λ production by wt and Siglec-H pDCs upon CpG stimulation, which is known to induce IFN-λ *via* TLR9. As controls we stimulated with LPS and R848, that act *via* TLR4 or TLR7, respectively, and are known not to induce IFN-λ (35). Interestingly, CpG stimulation did not induce a higher IFN-λ response in Siglec-H-deficient pDCs (**Figure 4A, B**). IFN-λcan also be triggered by the intracellular STING pathway and IRF3 (36). We therefore used 3’3’ cGAMP that stimulates the STING pathway and measured IFN-λ induction. Also by this stimulation there was no difference between wt and Siglec-H-deficient pDCs in IFN-λproduction (**Figure 4B**). Interestingly, 3’3’ cGAMP also induced a normal level of IFN-α in Siglec-H-deficient pDCs (**Figure 4B**). Therefore, it appears that Siglec-H selectively inhibits type I IFN responses by a TLR-9 dependent pathway on pDCs.

### Global Gene Expression Analysis of Siglec-H ko pDCs Reveals Downregulation of the HCK Kinase and the Chemokine Receptor CCR9, Which Affects pDC Migration

**Figure 5:**
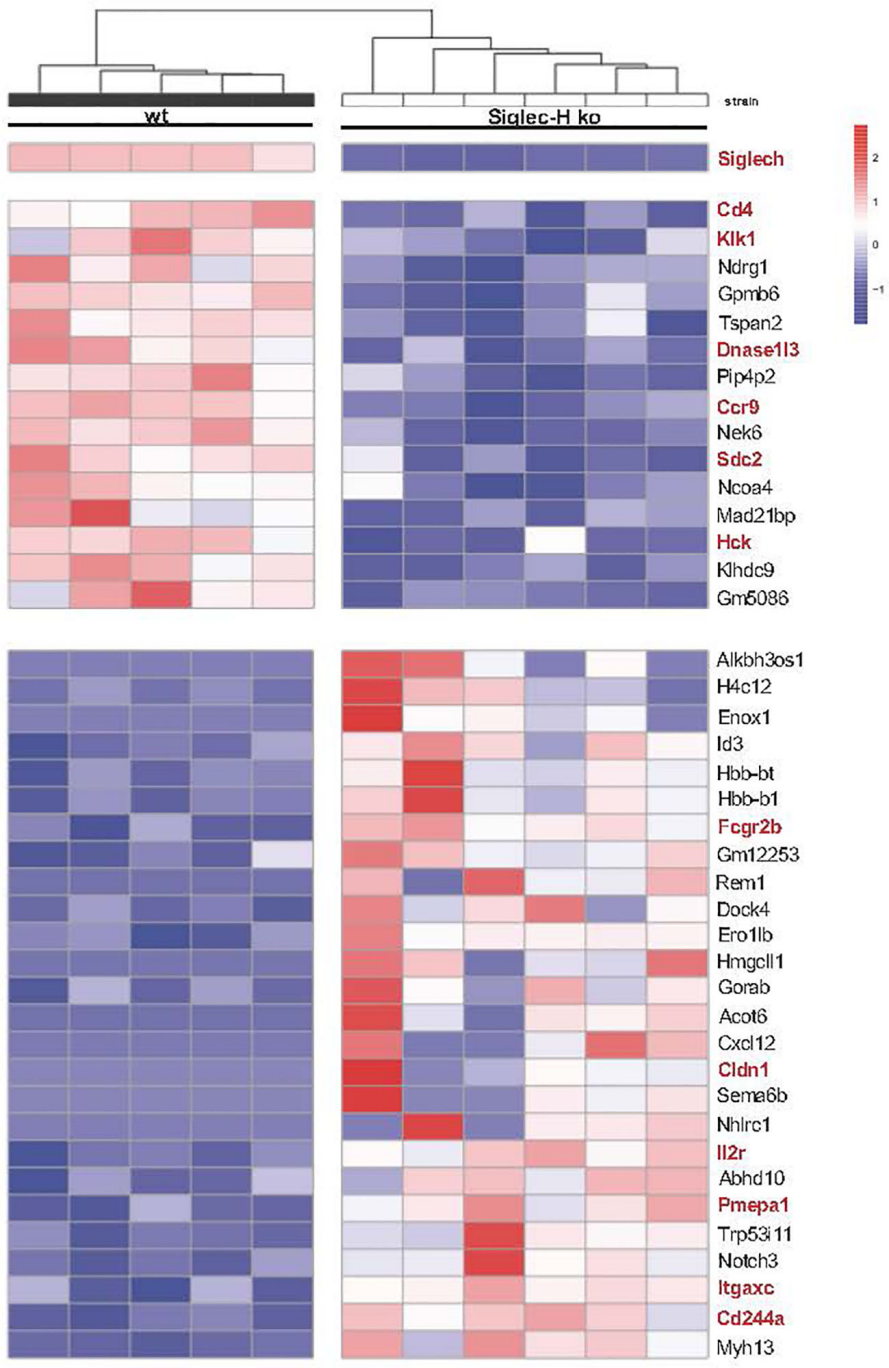
RNA-sequencing analysis of naive wt and Siglec-H ko pDCs. Naive splenic pDCs from wt or Siglec-H ko mice were sorted as shown in **Supplementary Figure 4B** (purity: around 90%) and RNA isolated. Bulk RNA-Sequencing of probes was done by NGS. All detected counts per sequence were normalised to total counts in each probe. Heatmap depicting genes with an adjusted p value<0,05. Interesting genes connected to immune cell functions, signaling and autoimmunity are marked red. Blue colour indicates reduced expression, red colour increased expression of genes. One experiment, N = 5-6 per genotype. Mice were aged 22-26w.

**Figure 6:**
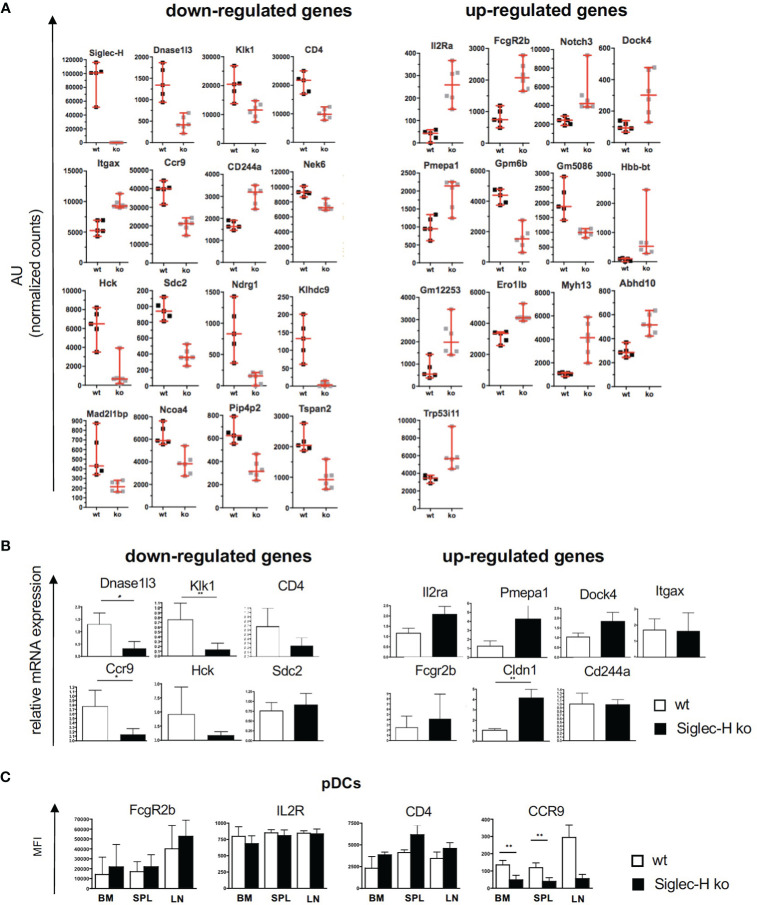
Overview of down- and up-regulated genes in naive splenic Siglec-H ko pDCs. **(A)** Scatter plots displaying the relative gene expression in wt and Siglec-H ko pDCs from RNA sequencing samples as shown in Figure 4. Sequence counts per gene were normalized to total counts per sample (AU= arbitrary units). Vertical bars indicate median, error bars are mean ± SD values. **(B)** pDCs from wt and Siglec-H ko mice were sorted and relative mRNA expression was verified *via* qRT-PCR. Bar charts show a summary of five independent experiments, n= 8-9. Mice were aged 8-14 weeks. Mann-Whitney test, *p < 0,05; **p < 0,01. Error bars are mean ± SD values. **(C)** Flow cytometric analysis of surface receptors on wt and Siglec-H ko pDCs in bone marrow (BM), spleen (SPL) and inguinal lymph nodes (LN). Bar charts display mean fluorescence intensity (MFI). Summary of two independent experiments with n=5. Error bars are mean ± SD values.

In order to determine the global influence of Siglec-H on signalling pathways and gene expression on pDCs, pDCs from spleens of wt and Siglec-H ko mice were sorted and analysed by bulk RNA sequencing analysis. RNA was isolated from pDCs of 5 wt and 6 Siglec-H ko animals to allow a good analysis of significantly regulated genes. The sorting strategy for splenic pDCs for this analysis is shown in **Supplementary Figure 4**. The results from the bulk RNA sequencing analysis were uploaded to the Geo server (https://www.ncbi.nlm.nih.gov/geo/) under the accession number GSE179840. An analysis focussing on significant differences (adjusted p-value ≤ 0.05) excluding genes, which were only expressed in at least half of the samples, identified 16 significantly downregulated and 26 significantly upregulated genes in Siglec-H-deficient pDCs that are shown in a heat map in **Figure 5**. Scatter plots showing all downregulated genes and 13 upregulated genes with levels that were at least at 20 arbitrary units (normalised counts), i.e. showed an expression level about a set threshold, are shown in **Figure 6A**. Of these differentially expressed genes several interesting candidates were followed up and their expression levels were verified by RT-qPCR. Interesting candidates were genes of cell surface proteins with known functions (*Cd4, Ccr9, Sdc2, Il2ra, Itgax, Fcgr2b, Cldn1, Cd244a*), intracellular signalling proteins (*Hck, Pmepa1 and Dock 4*) as well as genes linked to autoimmunity (*Dnase1l3* and *Klk1*). By qRT-PCR we could verify downregulation of *Dnase1l3, Klk1* and *Ccr9* and in tendency *Cd4* and *Hck* (**Figure 6B**). Among the upregulated genes we could just verify *Cldn1* and saw tendencies of upregulation for *Il2ra, Pmepa1, Dock4 and Fcgr2* (**Figure 6B**). Some of the differentially regulated cell surface proteins were also followed up by flow cytometry analysis of cell surface expression on pDCs of 3 different organs, as their functions are related to expression on the plasma membrane. The FcγRIIb inhibitory Fc receptor, IL2Rα (CD25) or CD4 were not expressed differentially on the surface of pDCs of Siglec-H ko mice, when compared to wt mice. However, the chemokine receptor CCR9 was downregulated on pDCs isolated from three different organs of Siglec-H ko mice (**Figure 6C**).

**Figure 7:**
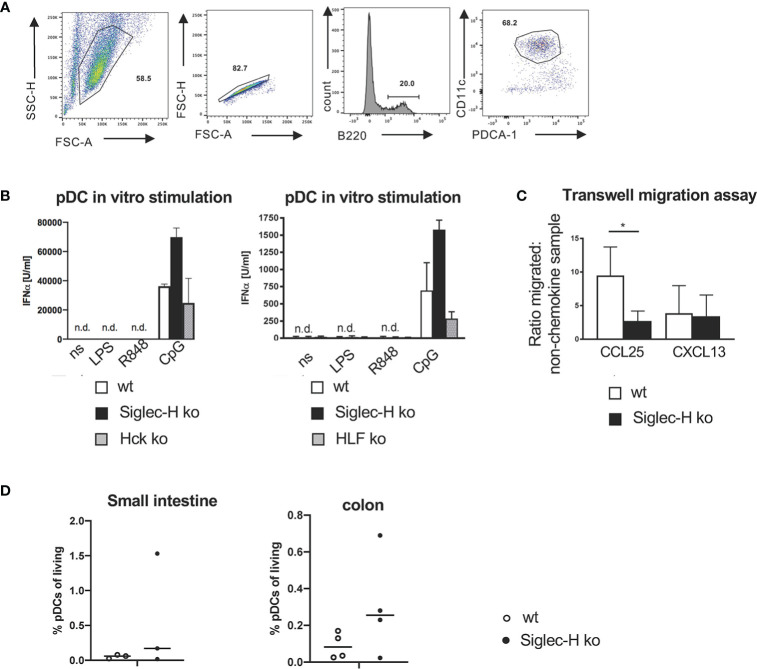
No influence of Hck in pDCs on the IFN-α production and reduced *in vitro* migration of Siglec-H ko pDCs to CCL25. **(A)** Strategy to sort pDCs from B16Flt3L-treated wt and Siglec-H ko spleens to use in transwell-migration assays (purity more than 90%). **(B)** *In vitro* generated pDCs from wt, Siglec-H ko and HCK ko or HFL KO mice were stimulated for 24 hrs with LPS (c=1 µg/ml), R848 (c=4 µg/ml) or CpG A (c=2,5 µg/ml) (two separate experiments are shown). The amount of IFN-α was measured by ELISA. White bars: wt, black bars: Siglec-H ko, grey bars: HCK ko (left), HFL ko (right). n.d.: not detectable. N=3 mice per genotype. Error bars are mean ± SD values. **(C)** Sorted pDCs were used in a transwell-migration assay to evaluate migratory capacity towards CCL25. CCL25 or CXCL13 were given in the lower chamber of the transwell and sorted pDCs were given into the upper chamber. pDCs could then migrate towards CCL25 or CXCL13 for 2 hrs. Migrated cells were counted in the lower chamber and a ratio in relation to the sample with no-chemokine in the lower chamber was calculated. As a negative control CXCL13 was used. CCL25 c= 125nM, CXCL13 c= 100nM. Ratio of migrated cells to non-chemokine control is displayed. Summary of three independent experiments with N=5 mice per genotype. Mice were aged 10-16 weeks. Mann-Whitney test, *p < 0,05. Error bars are mean ± SD values. **(D)** Leukocytes from the small intestine and bowel were isolated and stained as shown in **Supplementary Figure 4C**. pDC percentages are shown as % of living leukocytes. Mice were aged 10-16 weeks, N=3 mice per genotype.

Of these differentially expressed genes we first decided to follow up the downregulated *Hck* gene. Hck is a kinase of the Src family. Src family kinases are not only involved in activatory, but also in inhibitory pathways in immune cells (37, 38). If Hck would be critically involved in the inhibitory pathway of Siglec-H on type I IFN production, we would expect a similar increase of IFN-α after CpG stimulation of Hck-deficient pDCs. We generated pDCs from bone marrow of Hck ko mice and compared their response to wt and Siglec-H KO pDCs after CpG stimulation. While Siglec-H-deficient pDCs showed the increased IFN-α response, the IFN-α response of Hck-deficient pDCs was not increased (**Figures 7A, B**). As Hck has redundant functions to other expressed Src kinases in myeloid cells, the Hck function may be compensated by other Src members. Therefore, pDCs were generated from bone marrow of Hck/Fgr/Lyn triple-ko mice (29). and their response to CpG was analysed. Also triple-ko pDCs did not show an increased IFN-α response, in comparison to wt pDCs, the response was rather lower (**Figure 7B**). We therefore conclude that Hck or related Src kinases in pDCs are not directly involved in the Siglec-H dependent inhibitory pathway on IFN-α.

The second interesting differentially expressed gene is coding for the chemokine receptor CCR9, as it has important functions on pDCs (39) and is clearly downregulated on Siglec-H pDCs. We tested the functional consequence of the downregulation of CCR9 by a transwell migration assay. Siglec-H-deficient pDCs could migrate significantly less to the chemokine CCL25 that is a ligand for CCR9, but not to a control chemokine CXCL13 that binds to the chemokine receptor CXCR5 (**Figure 7C**). Thus, the downregulation of CCR9 on Siglec-H-deficient pDCs has functional consequences for these cells. CCR9 has been shown to be involved in pDC migration to the small intestine (39), therefore we analysed pDCs in the small intestine and colon of Siglec-H ko mice, but could not detect differences in cell numbers, when compared to wt mice (**Figure 7D**).

## Discussion

We studied the role of Siglec-H in mice using three different types of virus infections. Previously, we showed that mCMV infections of Siglec-H ko mice can trigger autoimmunity in a type I IFN-dependent manner. Siglec-H ko mice developed a severe autoimmune disease after mCMV infection, accompanied by a type I IFN signature (19). We could also show that Siglec-H-deficient pDCs produce higher IFN-α levels after TLR9 stimulation. This led to the model that Siglec-H is an inhibitory receptor controlling type-I IFN responses. Uncontrolled type-I IFN responses after a virus infection can then trigger a chronic inflammation leading to autoimmune disease. Here we show that neither influenza virus, nor chronic LCMV infections can trigger a similar type of autoimmunity and did not lead to signs of autoimmune disease in Siglec-H ko mice. This raises the question how these different outcomes of various virus infections in Siglec-H ko mice can be explained.

DNA viruses like CMV can enter pDCs *via* endocytosis and stimulate TLR9 *via* unmethylated CpG motifs in their DNA leading to IRF7-dependent stimulation of type I IFN genes. TLR9 responses are crucial for control of CMV infections, as mice with a mutated, non-functional TLR9 gene are highly susceptible to mCMV infections and show impaired type I IFN production (40). Siglec-H inhibits TLR9 responses on pDCs (19, 24). In contrast to other Siglecs, Siglec-H does not carry an inhibitory ITIM motif in its intracellular tail. However it interacts *via* a conserved lysine residue in the transmembrane domain to the adaptor protein DAP12 on the membrane of pDCs. DAP12-deficient pDCs lack Siglec-H membrane expression and generate higher amounts of IFN-α after CpG stimulation (27). This demonstrates the inhibitory role of DAP12 in these cells in response to TLR ligands. Siglec-H seems to be involved in the same pathway. Interestingly, Siglec-H only inhibits TLR9 dependent type I IFN production. IFN-α was increased only after CpG stimulation of Siglec-H-deficient pDCs, but not after stimulation of the intracellular STING pathway, as we show here in this manuscript. The negative regulation of TLR9-induced IFN-α responses by pDCs is in contrast to an unchanged IFN-α response of Siglec-H-deficient pDCs to TLR7 ligands (19). Both influenza virus and LCMV virus are ssRNA viruses that stimulate TLR7, instead of TLR9. For influenza it has been shown that endosomal recognition of influenza RNA is required for IFN-α response by pDCs (41). Similarly, LCMV genomic RNA also induces TLR7 responses (42). Since TLR7 stimulation of Siglec-H-deficient pDCs *in vitro* does not lead to increased IFN-α responses, this is one explanation why these two types of RNA viruses did not induce autoimmunity in Siglec-H ko mice.

CMV is a persistent virus. It causes 75% - 100% seroprevalence in wild mice and in the human population (43, 44). After infection of mice, a transient and immune-controlled reactivation of mCMV could constantly trigger low level of type I IFN production, which in the case of the Siglec-H ko mouse then leads to a chronic inflammatory milieu inducing a severe lupus-like autoimmune disease. Virus-induced IFN-α can lead to upregulation of TLRs on B cells and due to increased cell death, intracellular antigens, including DNA and RNA are released. These can be taken up as immune complexes into pDCs and can stimulate endosomal TLR7 or TLR9 receptors. This would lead to an enhancing feed-forward loop of inflammation inducing a lupus-like disease (7). All these processes cannot happen in influenza infections, as influenza causes an acute infection, which is rapidly cleared by the immune system (45). This would limit our findings to persistent viruses like CMV. For LCMV infections we chose clone 13, which is the persistent variant of the acute strain Armstrong (31). Clone 13 carries 2 amino acid exchanges in the virus surface protein GP1 and one exchange in the virus L polymerase. The mutations in GP1 lead to a higher affinity binding to its receptor on dendritic cells, altering DC cell functions leading to viral persistence. Similarly, the mutation in the L polymerase leads to a cellular replication advantage (32, 46). LCMV clone 13 causes functional exhaustion of CD8+ and CD4+ T cells (47, 48) and the immune systems gains only partial control of the virus. While the titers usually decline over times in blood and lymphoid organs and can become undetectable at ~4-8 weeks post infection, the virus remains in the mice and can at all times be detected in the kidneys (32, 49). It was shown that LCMV clone 13 also infects pDCs and downregulates their type I IFN response leading to an anergic state of pDCs (50). In contrast to mCMV, LCMV uses different mechanisms to promote its persistence such as T cell exhaustion. A downregulation of several immune cell functions by LCMV clone 13 may therefore not induce and maintain an inflammatory milieu, as it was observed after mCMV infections of Siglec-H ko mice. Nevertheless, the chronic LCMV infection induced quite high levels of autoantibodies several weeks after infection, both in wt and Siglec-H ko mice, but without any signs of kidney pathology.

Type III IFNs (IFN-λ1, IFN-λ2 and IFN-λ3) are important cytokines in antiviral immune responses. Increased levels of IFN-λ have also been demonstrated in SLE patients, with the highest levels found in patients with renal involvement and arthritis. IFN-λ also correlated with disease activity (33, 34). While several immune cell types can produce type III IFNs, among them pDCs, a functional IFN-λ receptor is restrictedly expressed and among the few immune cell types pDCs express it. This indicates that pDCs can produce these cytokines, but can also respond to them and data indicate that IFN-λ positively regulates several pDC functions (15). Therefore, it is of interest that Siglec-H does not inhibit IFN-λ responses, neither after intracellular STING-dependent activation nor after TLR9 stimulation. Thus, the inhibition of Siglec-H seems to be quite specific for TLR9-induced IFN-α responses.

DAP12 is associated to Siglec-H and seems to be involved in the inhibitory function of Siglec-H on pDCs. Both DAP12-deficient pDCs and DAP12-deficient macrophages produce more IFN-α or proinflammatory cytokines (27, 51). The mechanism of how DAP12 inhibits TLR signalling is unclear and is discussed in several models (26). While most Siglecs recognize sialic acids in forms and linkages that are commonly found on cell surfaces, the ligand for Siglec-H is not clear. Although the first Ig-domain of Siglec-H carries all the characteristic features for sialic acid binding, no binding to sialic acids had been detected so far by various attempts (22). Recently, Siglec-H binding to branched sialylated N-glycans isolated from mouse brain was demonstrated (52), which may be relevant for Siglec-H on microglia, the significance of this finding for mouse pDCs is unclear. Siglec-H does not have a clear orthologue in the human, but potential functional homologues with similar structures are Siglec-14 or Siglec-16 (19). We plan to analyse a possible involvement of Siglec-14 on human autoimmune diseases in the future.

When analysing the gene expression pattern of Siglec-H-deficient pDCs we expected to find differentially regulated genes of the type I IFN pathway. This is because of the observed strong upregulation of such genes after mCMV infection of Siglec-H ko mice. In the previous study we did not find any upregulation of type I IFN genes in spleen cells of uninfected mice, however (19). Nevertheless, Siglec-H ko mice develop some signs of autoimmunity when they age, however, without clear disease parameters. So the induction of this group of genes could only happen after a strong TLR9 stimulation. We will follow this up in the future. Differentially expressed genes between Siglec-H-deficient and wt pDCs were not found in large numbers, which may be due to the stringent analysis done with 5 and 6 mice per group. So we concentrated only on significantly changed gene expressions.

Among the three differentially expressed genes related to cell signalling processes we concentrated on Hck, a Src kinase, which was downregulated on Siglec-H-deficient pDCs. The related Src kinase Lyn is responsible for phosphorylation of inhibitory receptors such as FcgRIIb and CD22 (Siglec-2) (53, 54) in lymphocytes and Lyn KO mice develop autoimmunity (55). Therefore, it was of interest, whether Hck might also have a similar effect on Siglec-H or might be involved in the Siglec-H signalling pathway in pDCs. Hck-deficient pDCs did not show an enhanced CpG-induced IFN-α response. We also analysed Hck/Fgr/Lyn- triple KO pDCs, as these three Src kinases have redundant functions in myeloid cells (29), but also could not observe similarly enhanced IFN-α responses as in Siglec-H-deficient pDCs. Therefore, we can exclude that these kinases are directly involved in the Siglec-H/DAP12 signalling pathway. The downregulation of Hck on Siglec-H-deficient pDCs must therefore be a secondary effect, unrelated to the inhibition of type I IFN production.

Of several cell surface proteins that were differentially expressed between Siglec-H-deficient and wt pDCs, CCR9 was the only protein where a clear reduction of surface expression was detected as well. CCR9 is a chemokine receptor, which has been shown to be involved in pDC migration to the thymus and to the intestine (39). We did not detect any changes in pDCs numbers in the thymus (19) or in the small intestine. But these are findings are from mice at the steady state. CCL25-dependent migration into the small intestine of Siglec-H ko pDCs may be affected in inflammatory conditions such as colitis. This could be studied in the future. Furthermore, it was found that the CCR9+ subpopulation of pDCs produce less type I IFN than CCR9- pDCs (56) and CCR9+ pDCs can induce more immunosuppressive Tregs in graft-*versus*-host disease models (57). Here we did not observe any changes in Treg numbers after influenza or LCMV infections. But this may be different in autoimmune disease models where Tregs are crucial. This would also be interesting to study in the future.

Further differentially expressed genes of interest are Klk1 and DNase1l3, that are both associated with autoimmunity. Kallikrein-1 (Klk1) is a serine-protease which regulates blood pressure by acting on endothelium in blood vessels (58). Klk1 inhibits the transcription factor Irf7 that is crucial for expression of IFN−α and –β genes (59). As Klk1 is downregulated in Siglec-H-deficient pDCs, this is in line with the observed higher IFN-α production in Siglec-H ko mice. Klk1 mutations are also associated with lupus-nephritis in mice and humans, similar to the phenotype of Siglec-H ko mice (60). Finally Dnase1l3 is a secreted DNAse which is produced largely by myeloid cells and is thought to be involved in clearance of immune complexes containing DNA. DNase1l3-deficient mice develop autoantibodies and signs of a lupus-like autoimmune disease (61, 62). A downmodulation of DNase1l3 could contribute to the autoimmune phenotype of Siglec-H ko mice. We are crossing Siglec-H ko with Dnase1l3 ko mice at the moment to examine whether there are epistatic effects of these two genes in autoimmunity.

## Data Availability Statement

The datasets presented in this study can be found in online repositories. The names of the repository/repositories and accession number(s) can be found in the article/**Supplementary Material**.

## Ethics Statement

The animal study was reviewed and approved by Regierung von Unterfranken.

## Author Contributions

NS performed experiments and wrote the manuscript. OC, CL, HS, TC, AMa, and SC. JC and VB performed experiments. AMo, DZ, DD, and RH supervised the experiments. LN supervised the experiments and wrote the manuscript. All authors contributed to the article and approved the submitted version.

## Funding

This work was supported by the DFG through CRC1181 (project B06 to LN and A07 to DD) and RTG2504 (project B02 to CL and DD), Dutch Arthritis Association to RH and an ERC grant (ERC-2017-CoG - 772473 ToCCaTa) to DZ.

## Conflict of Interest

The authors declare that the research was conducted in the absence of any commercial or financial relationships that could be construed as a potential conflict of interest.

## Publisher’s Note

All claims expressed in this article are solely those of the authors and do not necessarily represent those of their affiliated organizations, or those of the publisher, the editors and the reviewers. Any product that may be evaluated in this article, or claim that may be made by its manufacturer, is not guaranteed or endorsed by the publisher.
