# Supplementary material for: Siglec-H-Deficient Mice Show Enhanced Type I IFN Responses, but Do Not Develop Autoimmunity After Influenza or LCMV Infections
Source: Front Immunol. 2021 Aug 23;12:698420. doi: 10.3389/fimmu.2021.698420 (PMC8419311; doi:10.3389/fimmu.2021.698420)
Supplement: Supplementary file 1 [file DataSheet_1.pdf]

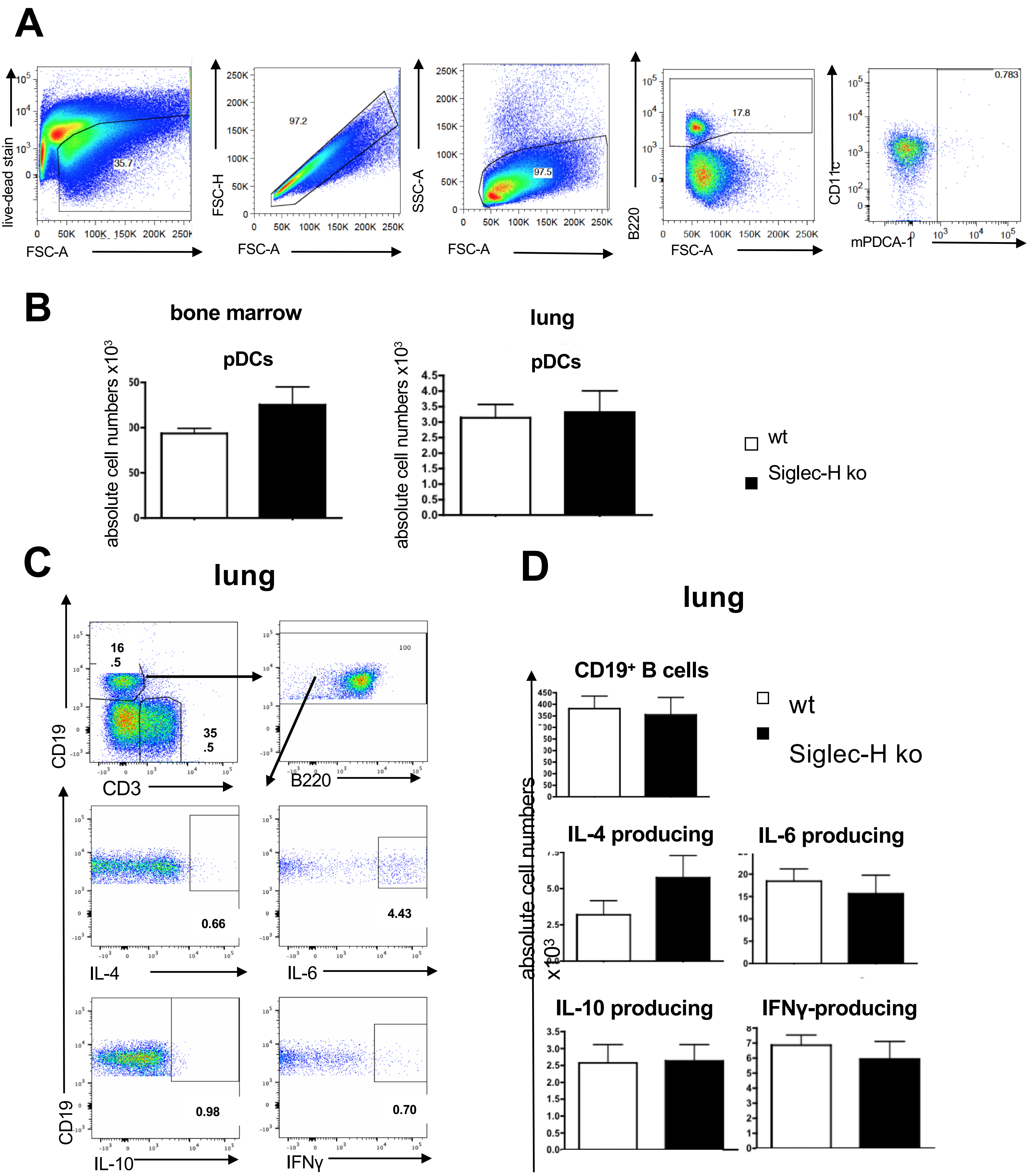

Supplementary Fig.1 No differences in pDC numbers nor in the cytokines produced by B cells in the lung
WT and Siglec-H KO mice were intranasaly infected with 1.5 x10^4 pfu Influenza H3N2 virus. Cell populations were analysed on day 10 p.i. (A) Gating strategy to gate pDCs in BM and lung. Single cells were stained on B220, CD11c and mPDCA-1. Cells positive for all three markers were gated as pDCs. (B) Bar charts showing pDCs in the BM and lung. (C) Gating strategy of intracellular cytokine staining of CD19+ B cells in the lung. (D) Bar charts showing the absolute cell numbers of CD19+ B cells producing different cytokines. White bar charts: WT, black bar charts: Siglec-H KO. Mice were aged between 8-14 weeks. Data from one experiment, N=10 per genotype. Error bars mark SD.

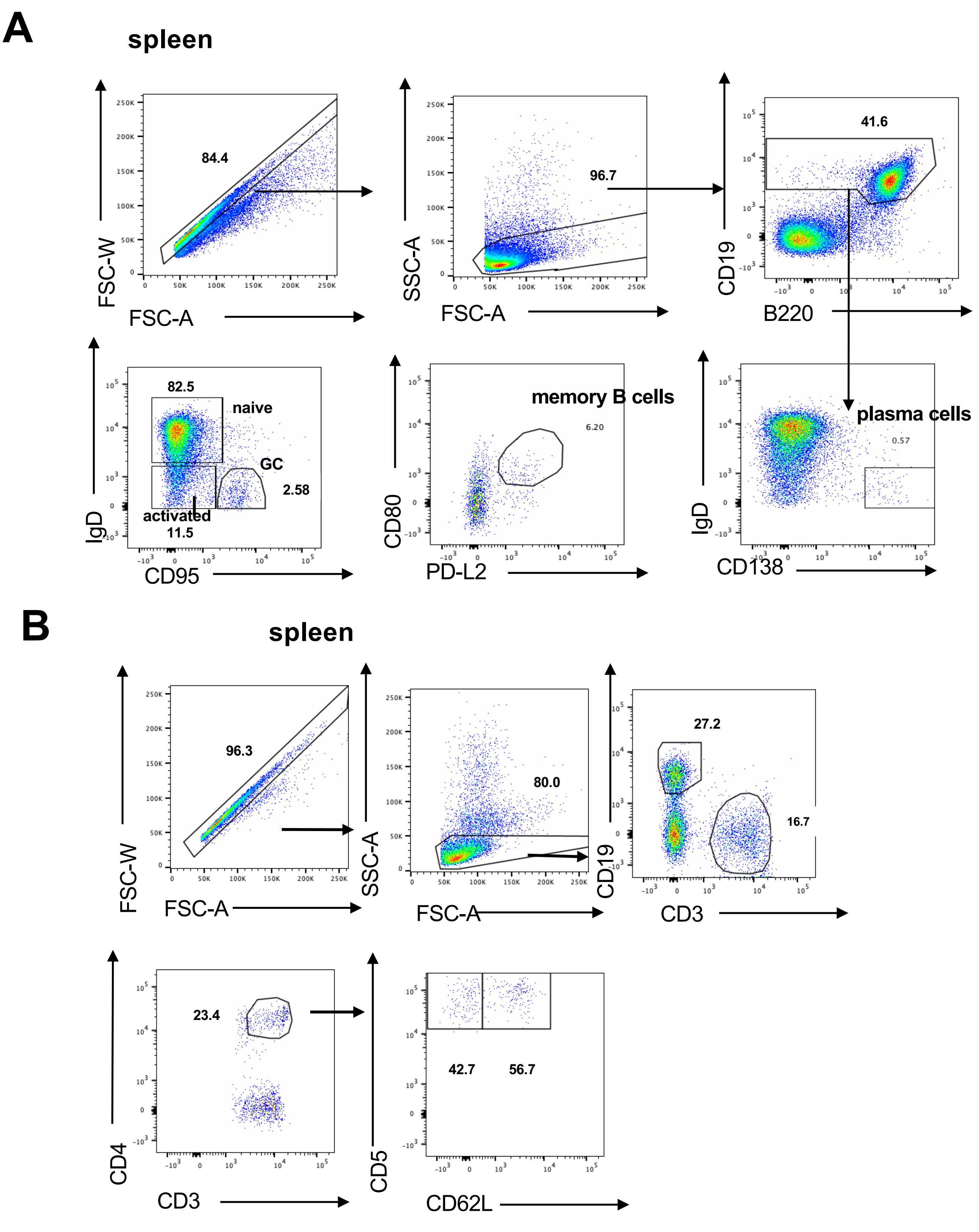

Supp. Figure 2: Gating strategy to analyse B cell and T cell subpopulations in lung and spleen 20 weeks after influenza infection.
WT and Siglec-H KO mice were intranasaly infected with 1.5 x10^4 pfu Influenza H3N2 virus. 20 weeks p.i. cell populations were analysed via flow cytometry. (A) Exemplary gating strategy for B cell subpopulations in the lung. CD19+ cells were stained with either CD80 and PDL2 for memory B cells or IgD and CD138 for plasma cells. The same strategy and markers were used to analyse B cells in the spleen. (B) Exemplary gating strategy for T cell subpopulations in the spleen. CD4+CD3+ T cells were divided into naive and activated T cells by CD62L surface expression. The same strategy and markers were used to analyse T cells in the lung.

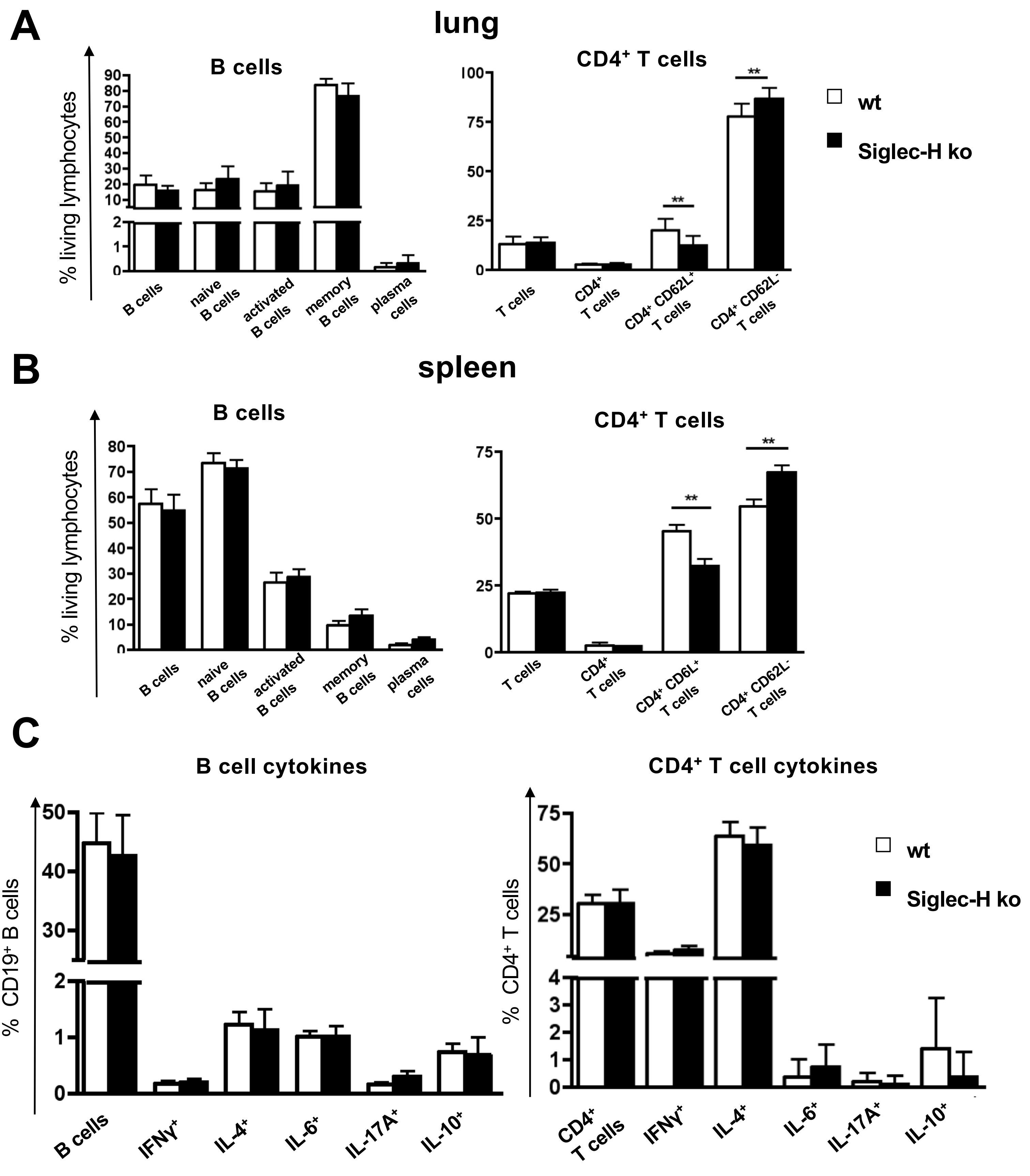

Supp. Fig. 3 Increased numbers of activated CD4+ T cells and no differences in B cell subpopulations.
WT and Siglec-H KO mice were intranasaly infected with 1.5 x10[4] pfu Influenza H3N2 virus. 20 weeks after infection the mice were euthanised and cell populations were analysed via flow cytometry in lung and spleen. (A-B) B cell subpopulations analysed in lung and spleen: naive B cells, activated B cells, memory B cells and plasma B cells (% of all B cells each). Analysed CD4+ T cell subpopulations were naive and activated T cells (% of CD4+ T cells) in lung and spleen. Shown are percent of living lymphocytes (C) Intracellular cytokines in CD19+B cells and CD4+ T cells in the spleen. White bars: wt, black bars: Siglec-H ko. One experiment was performed, N=10 mice. Mann-Whitney test, *P>0,05, **P<0,01. Error bars are mean ± SD values.

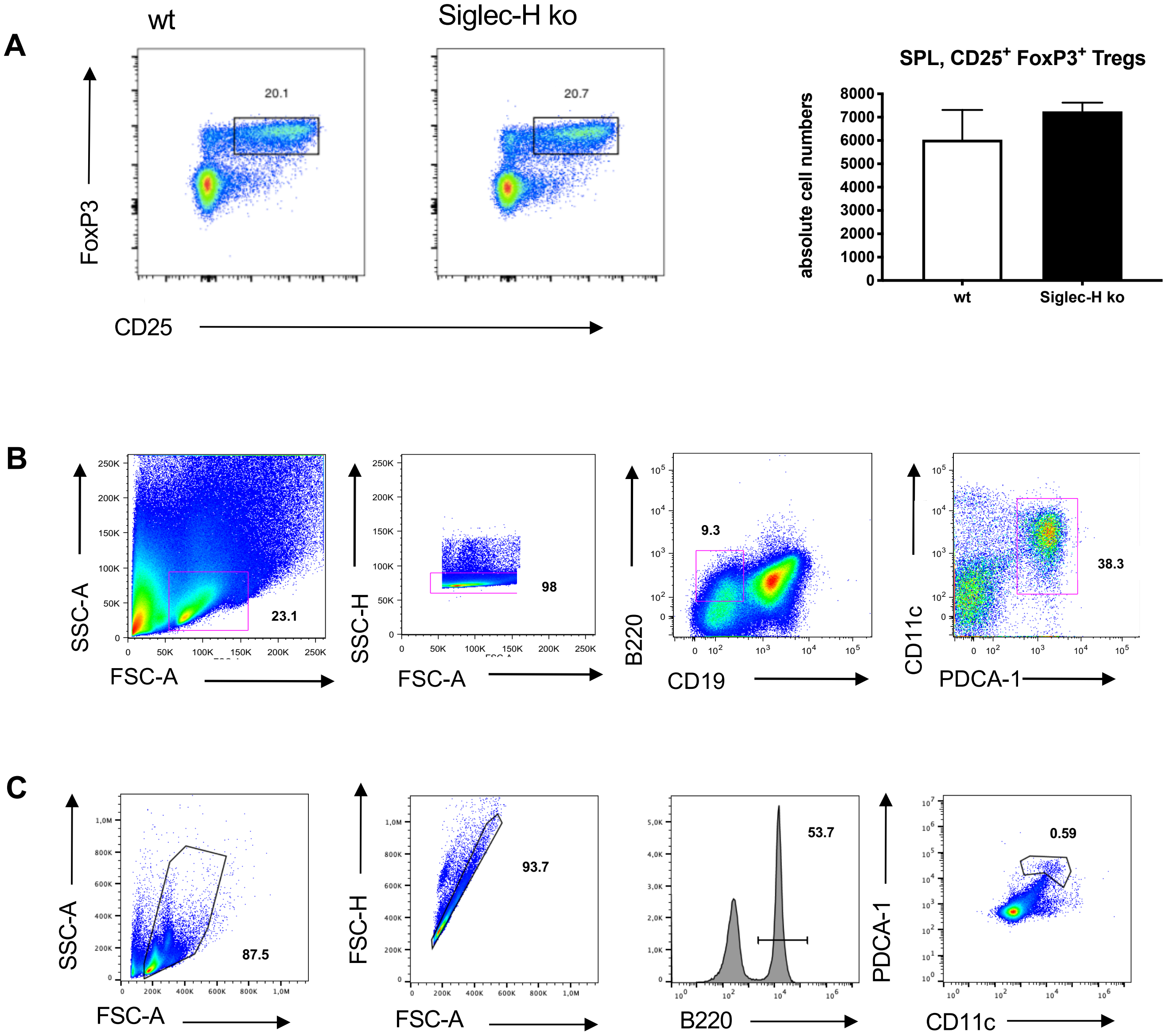

Supp. Fig. 4: Analysis of Tregs after LCMV infection and gating strategy to analyse pDCs.
(A) Analysis of FoxP3+, CD25+ Treg cells at day 25 after LCMV clone 13 infection (spleen)
(B) Exemplary gating strategy to sort pDCs from naive mice in the spleen for RNA-sequencing analysis. Spleen cells were stained with B220, CD19, CD11c and PDCA-1. B220+ CD19- CD11c int PDCA-1+ cells were sorted.(C) Exemplary gating strategy for flow cytometric analysis of pDCs in spleen. Single cells, which are B220+CD11c int PDCA-1+, were defined as pDCs. This strategy was used for bone marrow, lymph nodes and spleen.
